# Heart Rate Variability During Weaning from Invasive Mechanical Ventilation: A Systematic Review

**DOI:** 10.3390/jcm13247634

**Published:** 2024-12-15

**Authors:** Giovanni Giordano, Francesco Alessandri, Antonella Tosi, Veronica Zullino, Leonardo Califano, Luigi Petramala, Gioacchino Galardo, Francesco Pugliese

**Affiliations:** 1 Department of General and Specialistic Surgery, “Sapienza” University of Rome, 00185, Rome, Italy; francesco.alessandri@uniroma1.it (F.A.); califano.1922257@studenti.uniroma1.it (L.C.); f.pugliese@uniroma1.it (F.P.); 2 Department of Emergency, Critical Care and Trauma, Policlinico Umberto I, 00161, Rome, Italy; a.tosi@policlinicoumberto1.it (A.T.); v.zullino@policlinicoumberto1.it (V.Z.); luigi.petramala@uniroma1.it (L.P.); g.galardo@policlinicoumberto1.it (G.G.)

**Keywords:** autonomic nervous system, heart rate variability, spontaneous breathing trial, invasive mechanical ventilation, weaning

## Abstract

**Background:** The role of Heart Rate Variability (HRV) indices in predicting the outcome of the weaning process remains a subject of debate. The aim of this study is to investigate HRV analysis in critically ill adult patients undergoing weaning from invasive mechanical ventilation (IMV). **Methods:** The protocol of this systematic review was registered with PROSPERO (CRD42024485800). We searched PubMed and Scopus databases from inception till March 2023 to identify randomized controlled trials and observational studies investigating HRV analysis in critically ill adult patients undergoing weaning from invasive mechanical ventilation. Our primary outcome was to investigate HRV changes occurring during the weaning from IMV. **Results:** Seven studies (n = 342 patients) were included in this review. All studies reported significant changes in at least one HRV parameter. The indices Low Frequency (LF), High Frequency (HF), and LF/HF ratio seem to be the most promising in predicting the outcome of weaning with reliability. Some HRV indices showed modification in response to different ventilator settings or modalities. **Conclusions:** Available data report HRV modifications during the process of weaning and suggest a promising role of some HRV indices in predicting weaning outcomes in critically ill patients. Point-of-care HRV monitoring systems might help to early detect patients at risk of weaning failure.

## 1. Introduction

Among critically ill patients undergoing IMV, 10–40% experience failure in one or more weaning attempts, necessitating prolonged ventilator assistance [1]. Irrespective of the disease that prompted the patient’s admission to the Intensive Care Unit (ICU) and the necessity for IMV, ventilation weaning failure remains a negative prognostic factor, contributing to prolonged hospital length of stay and increased morbidity and mortality [2,3].

At present, the gold standard test to assess weaning readiness is the Spontaneous Breathing Trial (SBT) [4,5]. However, certain patients may successfully pass the SBT but subsequently fail weaning, requiring reintubation within the next 48 h [2]. The early detection of patients at higher risk of weaning failure is fundamental in order to reduce morbidity and to adopt adequate preventive measures.

The autonomic nervous system (ANS) regulates involuntary bodily functions, such as heart rate and respiratory rate, ensuring homeostasis through its sympathetic and parasympathetic branches, and by orchestrating responses in order to adapt to changing environmental demands [6]. As the transition from IMV to spontaneous breathing occurs, several physiological changes take place: intrathoracic pressure and pulmonary volumes change, resulting in hemodynamic alterations [7]. The ANS is activated, and it changes the heart rate (HR) through its sympathetic activity [6]. This is due to the close relationship between the respiratory and the cardiorespiratory centers, both of which are part of the ANS. The relationship between cardiovascular and pulmonary function (i.e., cardiopulmonary coupling), under the influence of the ANS, can help explain the influence of the latter during the process of liberation from IMV. Several studies have reported patients admitted to the ICU to be at risk of ANS dysfunction, and autonomic impairment proved to be of prognostic value in critically ill patients suffering from different disorders, including sepsis or intracerebral hemorrhage [8,9].

The HRV, defined as the variability of the R-R interval (RRi), has been proposed as a non-invasive marker of ANS activity and can be easily calculated with a 24-hour ECG and readily applied in clinical practice [10]. Growing evidence suggests a potential role of HRV during the weaning process; however, whether its use is beneficial in predicting the outcome of an SBT remains a subject of debate [11].

The aim of this review of literature is to investigate Heart Rate Variability analysis in critically ill adult patients undergoing weaning from IMV.

## 2. Materials and Methods

This systematic review was performed according to Preferred Reporting and Items for Systematic Review and Meta-Analyses recommendations. This study was preregistered on the PROSPERO database (ID CRD42024485800).

### 2.1. Search Strategy and Study Selection

Articles were screened by searching two different databases, namely, PubMed and Scopus, for clinical studies from inception till March 2023. We included studies classified as Clinical Randomized Trials, Clinical Trials, or Observational Studies that enrolled more than 10 patients. The following keywords were used for the search: «Heart Rate Variability and weaning from mechanical ventilation»; «HRV and weaning from mechanical ventilation»; «Autonomic system dysfunction and weaning from mechanical ventilation». The search strategy is reported in the Supplementary Materials section.

Two authors (GG and LC) selected articles that included critically ill adult patients undergoing IMV, either ready to be weaned or ready to receive a Spontaneous Breathing Trial, and were tested through HRV analysis to predict successful liberation from mechanical ventilation. Studies involving patients under 18 years of age, those with cardiac arrhythmias, individuals taking beta-blockers, those with hemodynamic instability, and those with altered mental status or under sedative drugs were excluded. After reviewing all articles, those not in English, duplicates, and irrelevant papers were also excluded. In cases of controversies between the two authors, a third opinion (FA) was sought; subsequently, the full-text versions of the selected articles were assessed.

The primary outcome of this study was to investigate HRV analysis changes in critically ill adult patients undergoing weaning from IMV. As a secondary outcome the efficacy of HRV indices to predict the successful weaning from IMV was investigated.

A standard form, in accordance with the ‘Cochrane Public Health Group’s Data Extraction and Assessment Template’, was designed and adapted for our Systematic Review to extract data from eligible studies. This form included information such as bibliographic sources, inclusion criteria, study design, risk of bias, analyzed HRV indices, study funding, and results.

### 2.2. Preplanned Strategy for Data Synthesis and Presentation

All selected studies were pooled together to evaluate the modifications of HRV indices during the weaning from IMV and to identify those associated with higher reliability to weaning success or failure. Results are displayed by reporting each different domain separately, as well as its modifications during the process of weaning and whether it can be associated with the weaning outcome. Statistical significance was considered at *p* < 0.05. Two authors were involved in the data synthesis (GG and FA), and a third opinion (LC) was sought in case of controversies between the two authors.

### 2.3. Risk of Bias and Quality of the Evidence

The risk of bias for randomized controlled trials (RCTs) was assessed using the Revised Cochrane risk-of-bias tool for randomized trials (RoB 2), structured into five domains: bias arising from the randomization process, bias due to deviations from intended interventions, bias due to missing outcome data, bias in the measurement of the outcome, and bias in the selection of the reported result [12]. For observational studies, the risk of bias was assessed with the “Risk Of Bias In Non-randomised Studies of Interventions” tool (ROBINS-I), which analyzes 7 domains [13,14]. The overall quality of the evidence for the primary outcome has been assessed according to Grading of Recommendations, Assessment, Development, and Evaluation (GRADE) guidelines [15,16,17]. Two authors (GG and LC) performed the analysis; a third opinion (FA) was requested in case of controversies between the two authors.

## 3. Results

**Figure 1:**
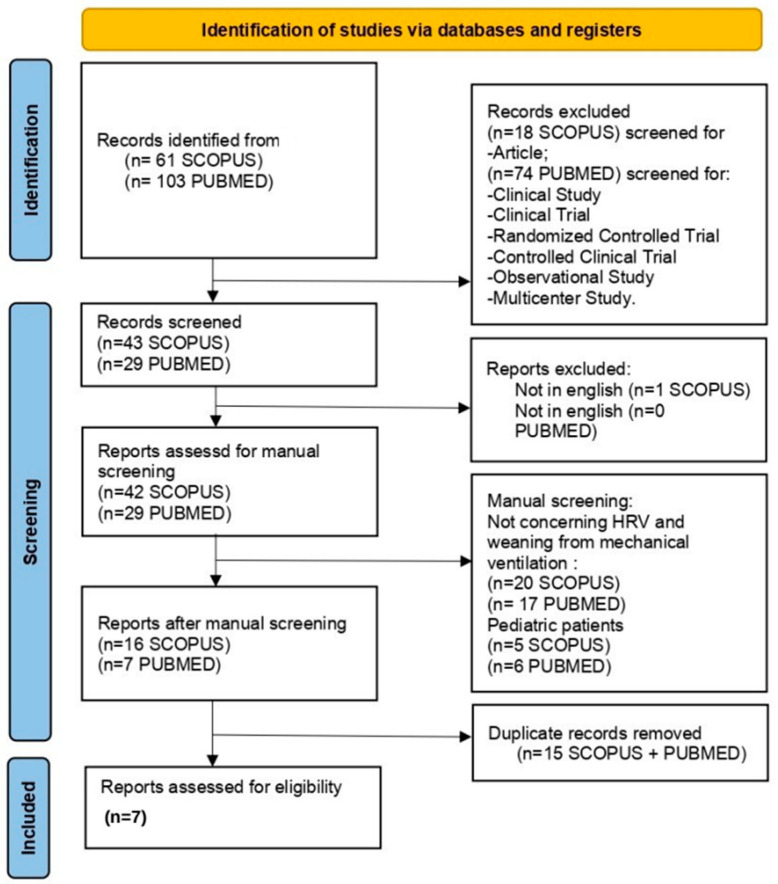
PRISMA 2020 flow diagram for new systematic reviews that included searches of databases and registers only.

A total of 164 studies resulted from the research; seven of them were eligible for this systematic review [10,11,18,19,20,21,22]. Among these, one was a randomized controlled trial (RCT), while the others were Prospective Observational studies (Figure 1, Table 1). An extended version of Table 1 is available in the Supplementary Materials section.

A total of 342 patients were included in this systematic review, ranging from a minimum of 18 to a maximum of 101. All studies were conducted in intensive care units (ICUs) and patients were not selected based on the disease that led to mechanical ventilation. Reported causes for IMV need included: acute hypoxemic respiratory failure, sepsis, acute respiratory distress syndrome, acute neurological critical illness, acute pancreatitis, cardio-respiratory arrest, and others. Common characteristics of patients included hemodynamic stability, resolution or improvement of the conditions leading to intubation, respiratory stability, and neurological stability. Five studies excluded tracheostomized patients.

In all the selected studies, enrolled patients underwent at least 24 h of IMV, some studies included patients after 48 h. Six studies had a pre-specified weaning protocol while one did not report any pre-planned strategy [18]. In different studies, SBT duration ranged from 10 min to 2 h. Spontaneous Breathing Trials were conducted using T-tubes in three studies [11,19,20] through a CPAP trial in one study [17], and with low-pressure support and PEEP [10,21] in two studies. In one case, patients received either PSV or T-tube (TT) SBT [22].

All studies reported HRV analysis before SBT, six studies during the SBT, and one after the SBT.

One study compared HRV indices in different consecutive modes of ventilation before performing TT-SBT [20].

Overall, all studies reported significant changes in at least one HRV parameter during the process of weaning from IMV (see Table 2, Table 3 and Table 4). A complete list of HRV indices, along with their abbreviations and short descriptions, is available in the Supplementary Material.

Moreover, some HRV indices have been shown to reliably predict successful (or unsuccessful) weaning. In particular, Low Frequency (LF), High Frequency (HF), and LF/HF ratio seem to be the most promising in predicting the outcome of weaning with reliability. However, some studies presented discordant and heterogeneous results.

Frequency domain measures (Table 2) were investigated in six studies and included LF (six studies), HF (six studies), Total Power (TP) (four studies) and LF/HF ratio (five studies), Very Low Frequency (VLF) (two studies).

Five of the six studies that investigated the LF parameter reported that this parameter significantly changes along the weaning process, during the SBT, compared to the pre-SBT. Of these, two studies reported an increase in LF; two studies reported an increase only in patients who failed the SBT, and one of them also reported a decrease in patients who successfully passed the SBT; one study reported a decrease in all patients. Four studies analyzed the differences among successfully or non-successfully weaned patients, reporting conflicting results. A higher value of LF before SBT was associated with weaning success in one study. Considering all studies pooled together, an increase in LF when passing from assisted ventilation to SBT is associated with higher odds of weaning failure.

HF showed changes during SBT in four out of six studies. One study reported a decrease during SBT while another reported an increase; three studies reported a decrease only in patients who failed the SBT, and one of them also reported an increase in the success group. Three studies analyzed the differences among successfully or non-successfully weaned patients, reporting conflicting results. A lower value of HF before SBT was associated with weaning failure in one study. Considering all studies pooled together, a decrease in HF when passing from assisted ventilation to SBT is associated with higher odds of weaning failure.

LF/HF was registered in five studies. Three out of five showed changes during the SBT of this parameter. In one study LF/HF increased only in the failure group and was higher before SBT compared to the success group; one study reported an increase only during TT-SBT when compared to PSV-SBT; one other showed a decrease during SBT.

Three studies reported TP variations during MV weaning. Among these, two studies reported a significant increase during the SBT in successfully weaned patients, and, in one of them, a decrease has been reported in patients who failed. A decrease during SBT in patients going to fail has been reported in one other study. One of these studies analyzed the TP value before SBT, reporting higher TP in successfully weaned patients.

The VLF parameter was not reported to significantly change, as it was reported to increase after SBT in successfully weaned patients in only one study, while the other two studies showed no significant changes. The first study also showed higher values of VLF in successfully weaned patients.

Time domain measures were investigated in four studies and included the standard deviation of all NN intervals (SDNN), the square root of the mean of the sum of the squares of differences between adjacent NN intervals (RMSSD), and others (Table 3).

SDNN was reported to change during the weaning in two studies out of four. One study showed an increase of this parameter during SBT in patients going to be successfully extubated only. In one study, it was reported to change when passing through different ventilation modes.

RMSSD has been reported to change in only one paper out of four: it showed an increase during SBT, but only in patients who successfully passed the SBT.

Patients included in the success group showed both SDNN and RMSSD scores significantly higher than in the failure group before SBT in two studies; however, this result was not confirmed in the two others.

SDNN index has been studied in one study only and was reported to increase from baseline to CPAP SBT in all patients. The other time domain parameters showed no significant changes during SBT.

Geometric domain measures were investigated in one study and included TINN, 10% WP, 50% WP, and TV (Table 4). This study showed an increase of 10% WP and 50% WP in the success group and a decrease of 10% WP in the failure group during CPAP-SBT, while TINN and TV did not significantly change.

### Risk of Bias and Quality of the Evidence

**Figure 2:**
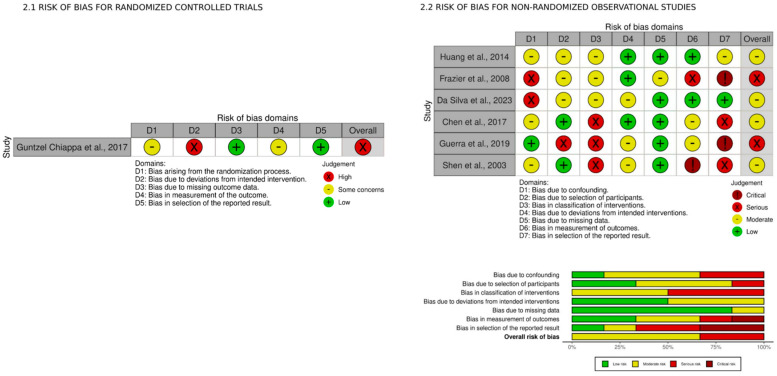
2.1. Risk of bias for randomized controlled trials. Guntzel Chiappa AM et al., 2017 [22]. 2.2. Risk of bias for non-randomized observational studies. Huang CT et al., 2014 [19]; Frazier SK et al., 2008 [18]; Da Silva RB et al., 2023 [10]; Chen YJ et al., 2017 [11]; Guerra M. et al., 2019 [20]; Shen HN et al., 2003 [21].

The risk of bias assessment for all included studies is reported in Figure 2. The included RCT shows a high risk of bias. Among non-RCTs, four show a moderate risk of bias, and two show a serious risk of bias, with the domains most frequently affected being ‘selection in the reported result’ and ‘classification of interventions’. The overall quality of evidence was rated as very low to low due to the limited number of studies, methodological limitations, and heterogeneity of results (Supplementary Materials).

## 4. Discussion

This is the first paper to systematically collect and report on the variation of HRV parameters during the process of liberation from IMV. Several HRV indices show modifications during the process of liberation from IMV, including LF, HF, LH/HF, TP, and SDNN. Heart Rate Variability tools may also have a role in helping clinicians predict the outcome of a weaning process, independently from the illness leading the patient to the ICU. Among the most promising indices, an increase in LF, as well as higher pre-SBT values, and/or a decrease in HF are associated with weaning failure. On the contrary, an increase in HF and/or a reduction in LF are associated with weaning success. Moreover, an increased value of LF/HF ratio during the SBT is associated with weaning failure and a reduced value is associated with weaning success.

No previous systematic review is available on this issue. A recent systematic review and meta-analysis identified twelve risk factors significantly associated with extubation failure, categorized into three domains: comorbidities, acute disease severity, and characteristics at the time of extubation [5]. Among these factors, heart rate and the rapid shallow breathing index (RSBI) were reported. Surprisingly, this paper did not consider HRV measures. However, results from this systematic review are consistent with the scarce evidence available. A previous paper showed that baseline HRV in critically ill patients is reduced when compared to normal values and that it further decreases during weaning [7]. It also showed a nine-fold increase in supraventricular beats during weaning and an HRV-predictive role of dysrhythmias. Other studies suggest that patients at risk of failing MV weaning have more severe cardiac and ANS dysfunction evaluated with HRV [21,23].

None of the studies included in this review investigated the predictive potential of combining different HRV indices together and/or with different classical parameters such as the rapid shallow breathing index or breathing variability [24]. A recent study conducted on a small group of patients found that including cardiopulmonary coupling indices in the weaning readiness criteria enhances the prediction of failing weaning attempts [25]. The study also noted that while some HRV parameters appear to differentiate patients likely to fail the SBT, concerns arise regarding the reliability of certain other HRV indices due to the potential influence of circadian rhythms. Another study observed that modifications in HRV and variability in respiration rate during the SBT correlate with weaning failure [26]. The review of respiratory rate variability (RRV) monitoring in weaning patients is beyond the scope of this manuscript; however, it is worth highlighting that the use of these additional indices may help complement the analysis of HRV. Incorporating RRV may enhance the prediction of successful weaning from mechanical ventilation by capturing dynamic interactions between the cardiovascular and respiratory systems.

In healthy individuals, the heart rate automaticity is under the control of the sinoatrial node, which is the natural pacemaker of the heart [27]. Despite this, the ANS continuously modulates cardiovascular and respiratory activity in response to various endogenous and exogenous stimuli to ensure efficient oxygen delivery in order to match metabolic demands and maintain homeostasis under different circumstances. The ANS also plays a pivotal role in regulating cardio-respiratory interactions: its sympathetic and parasympathetic branches exert dynamic control over heart rate, vascular tone, and respiratory rate. Sympathetic tone is enhanced during stress or exercise and increases heart rate and myocardial contractility. Concurrently, sympathetic activation drives pulmonary changes by increasing respiratory rate and inducing bronchodilation. Conversely, parasympathetic activity is prevalent during rest and digestion and promotes bradycardia, vasodilation, and bronchoconstriction. During the process of weaning, the changes in intrathoracic pressure and lung volume lead to hemodynamic alterations in the right and left ventricles preload and afterload, intrathoracic volume, and flow [28]. The ANS is therefore prompted to increase the sympathetic activity and/or to reduce the parasympathetic tone [21]. In healthy individuals, cardiopulmonary interactions are relevant in regulating autonomic balance, as it is demonstrated that positive pressure breathing enhances parasympathetic control of heart rate and alters hemodynamics [29,30]. However, a high incidence of ANS dysfunction has been reported in patients admitted to the ICU, potentially resulting in a poorer outcome and possibly to reduced likelihood of weaning success [31,32,33,34,35]. Moreover, poor cardiac function is reported to be a risk factor for failure of prolonged mechanical ventilation and weaning failure [36].

Heart Rate Variability, the beat-to-beat variation in a heartbeat, is a non-invasive marker of ANS activity that can be obtained by analyzing the variability of RRi in a standardized continued ECG registration, with the duration depending on the nature of the investigation [37]. When considering the ANS changing during various clinical situations, such as the weaning from mechanical ventilation, a registration time of at least 5–10 min is usually considered acceptable; however, longer records might also be useful [19,37]. Different parameters can be derived and are usually classified as frequency domain, time domain, or geometric domain measures. The HRV frequency domain analysis consists of a Fast Fourier Transform of the R-R interval time-series data and includes VLF (<0.04 Hz), LF (0.04–0.15 Hz), and HF (0.15–0.4 Hz) parameters. The absolute powers of VLF, LF, and HF are finally summed to calculate the TP. The HF measure reflects the parasympathetic activity, while LF reflects the sympathetic activity (21). However, there is no agreement among researchers concerning the real physiological meaning of LF, as well as of VLF [37]. The process of weaning may induce cardiopulmonary stress in particular patient groups. Specifically, individuals who fail SBTs experience elevated cardiovascular and pulmonary stress compared to those who successfully complete the trial [38]. A higher HF component in successfully weaned patients may be explained by a less stressful sympathetic activation led by the best respiratory activity. Conversely, when the cardio-respiratory system is unprepared, the weaning process becomes more taxing for the patient. This situation leads to heightened sympathetic activity and reduced vagal tone due to impaired physiological regulatory and adaptive mechanisms, resulting in increased LF values and decreased HF values [20,39,40]. In accordance with these findings, this systematic review reported significant changes in several HRV parameters during the process of weaning from MV, and that successfully weaned patients show an increase in HF values and/or a decrease in LF in most studies.

Moreover, some HRV indices showed modification in response to different ventilator settings or modalities. Recent evidence suggests that modalities that reduce ventilator dyssynchronies and the risk of over- or under-assistance are associated with higher rates of successful weaning, lower reintubation rates, and a shorter ICU length of stay [41]. Further studies should clarify the potential impact of different weaning modalities on ANS dysfunction.

Real-time analysis of HRV is not typically conducted in intensive care units (ICUs), despite the routine adoption of continuous electrocardiography monitoring; consequently, some authors suggest the development and implementation of point-of-care HRV monitoring systems [19]. This could be helpful to early detect patients at higher risk of weaning failure in order to adopt adequate measures, including the optimization of potentially reversible factors (e.g., cardiac, metabolic), performing a tracheostomy, implementing rehabilitation before the weaning attempt, the transfer toward specialized weaning units, or the decision to discharge the patient and start home ventilation [42].

This study has several limitations. First, it includes only a few papers, with only two of them being RCTs. Second, the lack of a pooled analysis might reduce the strength of the conclusions. Lastly, the risk of bias assessment for RCTs indicates some concerns, while non-RCTs show an overall moderate or serious risk of bias. The overall quality of evidence is rated as very low to low. However, this is the first systematic review to systematically record all available evidence on the use of HRV during the weaning from mechanical ventilation, and the reported data are homogeneous and robust enough to highlight the importance of ANS during the weaning process and to suggest a potential role of HRV in predicting the outcome of an SBT. More studies are needed in order to better clarify these important issues.

## 5. Conclusions

The role of the autonomic nervous system is crucial during weaning from mechanical ventilation, as it plays a pivotal role in regulating cardio-respiratory interactions and in response to the hemodynamic alterations occurring throughout the process. The high incidence of ANS dysfunction in critically ill patients might contribute to a reduced likelihood of passing the spontaneous breathing trial. Heart Rate Variability can be considered a non-invasive and easy-to-interpret parameter of ANS activity, although its routine use in clinical practice is uncommon. According to this systematic review, several HRV indices exhibit changes during liberation from invasive mechanical ventilation, including LF, HF, LF/HF ratio, TP, and SDNN. Furthermore, available data suggest a promising role in predicting weaning outcomes in critically ill patients. Among the most promising indices, an increase in LF or LF/HF ratio, as well as a decrease in HF, are associated with weaning failure. The implementation of point-of-care HRV monitoring systems might help to early detect patients at higher risk of weaning failure, enabling the adoption of adequate preventive measures. Further studies are needed to investigate the potential role of combining different HRV indices and/or classical parameters (e.g., RSBI or breathing variability) and the potential impact of different weaning modalities on ANS dysfunction.

## Figures and Tables

**Table 1:** Studies included in this systematic review.

Reference	Study Type	Subjects (*N),* Setting	Duration of MV	Exclusion Criteria	Weaning Protocol	SBT	HRV Measurement Timing
Da Silva RB et al., 2023 [10]	P Obs	68 ICU	24+	Tracheostomies, arrhythmias, cardiac pacemaker, heart transplant, taking antiarrhythmic drugs	YES	30 min PSV	Pre-SBT
Chen YJ et al., 2017 [11]	P Obs	67 ICU and RCU	24+	SBP < 90 mmHg, vasoactive or inotropic drugs, hyperthermia, hemoglobin ≤ 10 g/dL, high-carbohydrate diet, altered mental status	YES	2 h T-tube	Pre-SBT SBT
Frazier SK et al., 2008 [18]	P Obs	43 ICU	48+	Neuromuscular disease, terminal, cardiac pacemaker, experienced myocardial infarction or cerebrovascular accident within the past 6 months, receiving B-adrenergic antagonist drugs, tracheostomies	NO	2 h CPAP	− Pre SBT (During IMV) − During SBT (1hr prior to the initiation of the CPAP trial and continuing for 24 h)
Huang CT et al., 2014 [19]	P Obs	101 ICU	24+	Tracheostomies, arrhythmias, not cooperative, SBT less than 30 min, airway obstruction	YES	60 min T-tube	− Pre-SBT − SBT period − Postextubation period
Guerra M. et al., 2019 [20]	P Obs	18 ICU	24+	Not ready for weaning from IMV, vasopressor drug dependence, absence of respiratory drive and effective cough, coma, pH < 7, 30, abnormal values of serum Na, K, Ca, Mg	YES	10 min T-tube	A/C SIMV PSV 18 PSV 10 TT
Shen HN et al., 2003 [21]	P Obs	24 ICU	24+	Tracheostomies, frequent cardiac arrhythmias	YES	30 min PSV	Pre-SBT A/C Pre-SBT PSV SBT
Guntzel Chiappa AM et al., 2017 [22]	RCT	21 ICU	48+	Previous arterial hypotension, arrhythmias, cardiac pacemaker implantation, severe brain disease, barotrauma, presence of thoracic drain, tracheostomies, vasoactive or sedative drugs	YES	30 min PSV or T-tube	SBT TT SBT PSV

A/C = Assisted Controlled Ventilation; CPAP = Continuous Positive Airway Pressure; MV = Mechanical Ventilation; P OBs = Observational Prospective; RCT = Randomized Controlled Trial; RCU = Respiratory Care Unit; SBP = Systolic Blood pressure; SBT = Spontaneous Breathing Trial; SIMV = Synchronized Intermittent Mandatory Ventilation; PSV = Pressure Support Ventilation; TT = T Tube (T-piece).

**Table 2:** Outcomes reported in included studies, frequency domain.

Indices	Study	Results
VLF	Huang CT et al., 2014 [19]	− Higher before SBT in success group − Increased during SBT in success group
	Chen YJ et al., 2017 [11]	− No changes during SBT compared to pre-SBT in both groups.
LF	Huang CT et al., 2014 [19]	− No changes during SBT compared to pre-SBT in both groups. − No differences between failure and success groups in pre-SBT values.
	Guntzel Chiappa AM et al., 2017 [22]	Increase during SBT
	Guerra M. et al., 2019 [20]	Increase during SBT
	Da Silva RB et al., 2023 [10]	Increase during SBT in failure group only
	Chen YJ et al., 2017 [11]	− Increase in failure group and decrease in success group during SBT − Higher pre-SBT LF associated with weaning success compared to weaning failure
	Shen HN et al., 2003 [21]	− Decrease during SBT in failure group − No differences among failure and success group variations
HF	Huang CT et al., 2014 [19]	− No changes during SBT compared to pre-SBT in both groups. − No differences between failure and success groups in pre-SBT values.
	Guntzel Chiappa AM et al., 2017 [22]	Decrease during SBT
	Shen HN et al., 2003 [21]	− Decrease during SBT in failure group only
	Da Silva RB et al., 2023 [10]	− Decrease during SBT in failure group only − Lower in failure group before SBT compared to success group
	Guerra M. et al., 2019 [20]	− Increase in SIMV and PSV − No change during SBT
	Chen YJ et al., 2017 [11]	− Increase during SBT in success group and decrease in failure group − Higher values pre-SBT associated with successful weaning
LF/HF	Da Silva RB et al., 2023 [10]	− Increase during SBT in failure group only − Higher in failure group before SBT
	Guntzel Chiappa AM et al., 2017 [22]	Increase during TT-SBT
	Huang CT et al., 2014 [19]	No significant change
	Guerra M. et al., 2019 [20]	Decrease during SBT
	Shen HN et al., 2003 [21]	No significant change
TP	Huang CT et al., 2014 [19]	− Increase during SBT in successfully weaned patients − Higher in patients successfully weaned before SBT
	Chen YJ et al., 2017 [11]	Decrease during SBT in failure group and increase in success group
	Shen HN et al., 2003 [21]	Decrease in failure group during SBT

HF = High Frequency; LF = Low Frequency; SBT = Spontaneous Breathing Trial; SIMV = Synchronized Intermittent Mandatory Ventilation; PSV = Pressure Support Ventilation; TP = Total Power; TT = T Tube (T-piece); VLF = Very Low Frequency.

**Table 3:** Outcomes reported in included studies, time domain.

Indices	Study	Results
SDNN	Frazier SK et al., 2008 [18]	No significant change during SBT
	Da Silva RB et al., 2023 [10]	No significant change during SBT
	Chen YJ et al., 2017 [11]	Increase during SBT in successfully weaned patients only
	Guerra M. et al., 2019 [20]	Decrease during SBT
RMSSD	Frazier SK et al., 2008 [18]	No significant change
	Da Silva RB et al., 2023 [10]	No significant change
	Chen YJ et al., 2017 [11]	Increase during SBT in successfully weaned patients only
	Guerra M. et al., 2019 [20]	No significant change
SDANN	Frazier SK et al., 2008 [18]	No significant change
SDSD	Frazier SK et al., 2008 [18]	No significant change
SDNN index	Frazier SK et al., 2008 [18]	Increase during CPAP-SBT
Mean RRi	Da Silva RB et al., 2023 [10]	No significant change
pNN50	Guerra M. et al., 2019 [20]	No significant change

Mean RRi = Mean R-R interval; pNN50 = Percentage of successive differences in the R-R intervals, whose absolute value exceeds 50 ms; RMSSD = square root of the mean of the sum of squares of differences between adjacent normal R-R intervals; SDANN = standard deviation of means of R-R intervals of successive 5-min epochs over the 24-h data recording; SDNN = standard deviation of the normal R waves; SDNN index = mean of the standard deviation of all R-R intervals for all 5-min epochs captured; SDSD = standard deviation of the differences adjacent R-R intervals.

**Table 4:** Outcomes reported in included studies, geometric domain.

Indices	Study	Results
TINN	Frazier SK et al., 2008 [18]	No significant change
10% WP	Frazier SK et al., 2008 [18]	Increase during SBT in success group and decrease in failure group
50% WP	Frazier SK et al., 2008 [18]	Increase during SBT in successfully weaned patients
TV	Frazier SK et al., 2008 [18]	No significant change

10% WP = width of the histogram at 10% of the peak of the triangle; 50% WP = width of the histogram at the 50% of the peak of the triangle; TINN = width of the histogram distribution measured at the base of a triangle; TV = width of the histogram at the base.

## Supplementary Materials

The following supporting information can be downloaded at: https://www.mdpi.com/article/10.3390/jcm13247634/s1.
