# Supplementary material for: Heart Rate Variability During Weaning from Invasive Mechanical Ventilation: A Systematic Review
Source: J Clin Med. 2024 Dec 15;13(24):7634. doi: 10.3390/jcm13247634 (PMC11727775; doi:10.3390/jcm13247634)
Supplement: Supplementary file 1 [file jcm-13-07634-s001.zip › jcm-3354755-supplementary.pdf]

Heart Rate Variability during weaning from invasive mechanical ventilation: a systematic review.

Giovanni Giordano[1]*, Francesco Alessandri[1], Leonardo Califano[1], Luigi Petramala[2], Francesco Pugliese[1].

1. Department of General Surgery, Surgical Specialties and Organ Transplantation "Paride Stefanini", Sapienza University of Rome, Policlinico Umberto I, Rome, Italy.
2. Department of Translational and Precision Medicine, "Sapienza" University of Rome, Rome, Italy.

* Correspondence to: Giovanni Giordano, Department of General Surgery, Surgical Specialties and Organ Transplantation "Paride Stefanini", Sapienza University of Rome, Policlinico Umberto I, Rome, Italy. E-mail address: gi.giordano@policlinicoumberto1.com

Search Strategy for Medline

#1

Search: (Heart Rate Variability) AND (weaning from mechanical ventilation)

("heart rate"[MeSH Terms] OR ("heart"[All Fields] AND "rate"[All Fields]) OR "heart rate"[All Fields]) AND ("variabilities"[All Fields] OR "variability"[All Fields] OR "variable"[All Fields] OR "variable s"[All Fields] OR "variables"[All Fields] OR "variably"[All Fields]) AND (("weaning"[MeSH Terms] OR "weaning"[All Fields] OR "weaned"[All Fields] OR "weanings"[All Fields] OR "weans"[All Fields]) AND ("respiration, artificial"[MeSH Terms] OR ("respiration"[All Fields] AND "artificial"[All Fields]) OR "artificial respiration"[All Fields] OR ("mechanical"[All Fields] AND "ventilation"[All Fields]) OR "mechanical ventilation"[All Fields]))

Translations

Heart Rate: "heart rate"[MeSH Terms] OR ("heart"[All Fields] AND "rate"[All Fields]) OR "heart rate"[All Fields]

Variability: "variabilities"[All Fields] OR "variability"[All Fields] OR "variable"[All Fields] OR "variable's"[All Fields] OR "variables"[All Fields] OR "variably"[All Fields]

weaning: "weaning"[MeSH Terms] OR "weaning"[All Fields] OR "weaned"[All Fields] OR "weanings"[All Fields] OR "weans"[All Fields]

mechanical ventilation: "respiration, artificial"[MeSH Terms] OR ("respiration"[All Fields] AND "artificial"[All Fields]) OR "artificial respiration"[All Fields] OR ("mechanical"[All Fields] AND "ventilation"[All Fields]) OR "mechanical ventilation"[All Fields]

#2

Search: (HRV) AND (weaning from mechanical ventilation)

"HRV"[All Fields] AND (("weaning"[MeSH Terms] OR "weaning"[All Fields] OR "weaned"[All Fields] OR "weanings"[All Fields] OR "weans"[All Fields]) AND ("respiration, artificial"[MeSH Terms] OR ("respiration"[All Fields] AND "artificial"[All Fields]) OR "artificial respiration"[All Fields] OR ("mechanical"[All Fields] AND "ventilation"[All Fields]) OR "mechanical ventilation"[All Fields]))

Translations

weaning: "weaning"[MeSH Terms] OR "weaning"[All Fields] OR "weaned"[All Fields] OR "weanings"[All Fields] OR "weans"[All Fields]

mechanical ventilation: "respiration, artificial"[MeSH Terms] OR ("respiration"[All Fields] AND "artificial"[All Fields]) OR "artificial respiration"[All Fields] OR ("mechanical"[All Fields] AND "ventilation"[All Fields]) OR "mechanical ventilation"[All Fields]

#3

Search: (Autonomic system dysfunction) AND (weaning from mechanical ventilation)

("autonomic nervous system"[MeSH Terms] OR ("autonomic"[All Fields] AND "nervous"[All Fields] AND "system"[All Fields]) OR "autonomic nervous system"[All Fields] OR "autonomic"[All Fields] OR "autonomical"[All Fields] OR "autonomically"[All Fields] OR "autonomics"[All Fields]) AND ("system"[All Fields] OR "system s"[All Fields] OR "systems"[All Fields]) AND ("dysfunctional"[All Fields] OR "dysfunctionals"[All Fields] OR "dysfunctioning"[All Fields] OR "dysfunctions"[All Fields] OR "physiopathology"[MeSH Subheading] OR "physiopathology"[All Fields] OR "dysfunction"[All Fields]) AND (("weaning"[MeSH Terms] OR "weaning"[All Fields] OR "weaned"[All Fields] OR "weanings"[All Fields] OR "weans"[All Fields]) AND ("respiration, artificial"[MeSH Terms] OR ("respiration"[All Fields] AND "artificial"[All Fields]) OR "artificial respiration"[All Fields] OR ("mechanical"[All Fields] AND "ventilation"[All Fields]) OR "mechanical ventilation"[All Fields]))

Translations

Autonomic: "autonomic nervous system"[MeSH Terms] OR ("autonomic"[All Fields] AND "nervous"[All Fields] AND "system"[All Fields]) OR "autonomic nervous system"[All Fields] OR "autonomic"[All Fields] OR "autonomical"[All Fields] OR "autonomically"[All Fields] OR "autonomics"[All Fields]

system: "system"[All Fields] OR "system's"[All Fields] OR "systems"[All Fields]

dysfunction: "dysfunctional"[All Fields] OR "dysfunctionals"[All Fields] OR "dysfunctioning"[All Fields] OR "dysfunctions"[All Fields] OR "physiopathology"[Subheading] OR "physiopathology"[All Fields] OR "dysfunction"[All Fields]

weaning: "weaning"[MeSH Terms] OR "weaning"[All Fields] OR "weaned"[All Fields] OR "weanings"[All Fields] OR "weans"[All Fields]

mechanical ventilation: "respiration, artificial"[MeSH Terms] OR ("respiration"[All Fields] AND "artificial"[All Fields]) OR "artificial respiration"[All Fields] OR ("mechanical"[All Fields] AND "ventilation"[All Fields]) OR "mechanical ventilation"[All Fields]

Supplementary Table S1. Studies included in this Systematic Review. Extended version.

REF	SUBJECTS, *N*	HOURS OF IMV	EXCLUSION CRITERIAS	WEANING PROTOCOL	SBT	HRV MEASUREMENT TIMING	MODE AND DOMAIN OF ANALYSIS	RESULTS PRE SBT	RESULTS SBT	RESULTS Δ SBT / POST EXTUB
Huang Chun-Ta et al. P Obs 2014	101 ICU	24+	Tracheostomies, arrythmias, not cooperative, SBT less than 30 min, airway obstruction	YES	60 min T-tube	-Pre-SBT -SBT period - Postextubation period	*Frequency Domain Measures:* -VLF -LF -HF -TP -LF/HF	*Frequency Domain Measures:* Higher VLF and TP in successfully weaned patients	Δln TP ≤0.4 ln(ms2) was associated with an odds ratio (OR) of failed SBT of 3.1 (95% confidence interval (CI), 1.2 to 8.0).	Δln VLF <0.1 ln(ms2) (OR, 3.9; 95% CI, 1.1 to 14.8) and Δln TP <0.02 ln(ms2) (OR, 6.6; 95% CI, 1.6 to 27.9) remained significantly related to development of extubation failure.
Frazier S. K. et al. P Obs 2008	43 ICU	48+	Neuromuscular disease, terminal, cardiac pacemaker, experienced myocardial infarction or cerebrovascular accident within the past 6 months, receiving B-adrenergic antagonist drugs, tracheostomies	NO	2 hours CPAP	-Pre SBT (During IMV) -During SBT (1hr prior to the initiation of the CPAP trial and continuing for 24 h)	*Time Domain Measures:* -SDNN -RMSSD -SDANN -SDSD -SDNN index *Geometric Domain Measures:* -TINN -10% WP -50% WP -TV	*Time Domain Measures:* Patients in the FG had statistically significantly lower values of SDNN, RMSSD, and SDSD, compared with patients in SG	*Time Domain Measures:* No differences between the groups during the CPAP trial *Geometric Measures:* 10% and 50% WP increased during CPAP trial in SG decreased in FG. TV was unchanged in SG and reduced in FG	
Da Silva R. B. et al. P Obs 2023	68 ICU	24+	Tracheostomies, arrythmias, BAV II/III, cardiac pacemaker, heart transplant, taking antiarrythmic drugs	YES	30 min PSV	Pre-SBT	*Time Domain Measures:* -Mean RRi -SDNN -RMSDD *Frequency Domain Measures:* -LF -HF -LF/HF	*Time Domain Measures:* No statistical differences *Frequency Domain Measures:* Incres		

								e in LF and LF/HF and decrease in HF in FG		
Guntzel Chiappa A. M. et al. RCT 2015	21 ICU	48+	Previous arterial hypotension, arrythmias, cardiac pacemaker implantation, severe brain disease, barotrauma, presence of thoracic drain, tracheostomies, vasoactive or sedative drugs	YES	30 min PSV or T-tube	SBT TT SBT PSV	*Frequency Domain Measures:* -TP -LF -HF -LF/HF		*Frequency Domain Measures:* LF and LF/HF increased during TT compared with PSV and HF decreased	
Yu-Ju Chen et al. P Obs 2017	67 ICU and Respiratory Care Unit	24+	SBP < 90 mmHg, vasoactice or inotropic drugs, hyperthermia, hemoglobin ≤ 10 g/dL, high-carbohydrate diet, alterated mental status	YES	2 hours T-tube	Pre-SBT SBT	*Time Domain Measures:* -SDNN -RMSSD *Frequency Domain Measures:* -VLF -LF -HF -TP	*Time Domain Measures:* No significantly differences between FG and SG *Frequency Domain Measures:* In LF was significantly higher in the success group than in the failure group	*Time Domain Measures:* SDNN and RMSSD increased in SG and not in FG *Frequency Domain Measures:* HF increased in SG and decreased in FG; LF decreased in SG and increased in FG	
Guerra M. et al. P Obs 2019	18 ICU	24+	Not ready for weaning from IMV, vasopressor drug dependence, absence of respiratory drive and effective cough, not responsive to sound stimuli, pH <7,30, abnormal values of	YES	10 min T-tube	A/C SIMV PSV 18 PSV 10 TT	*Time Domain Measures:* -RMSSD -pNN50 -SDNN *Frequency Domain Measures:* -HF -LF LF/HF		LF component increased in the different ventilatory types in relation to the spontaneous respiration through the TT (A/C to TT; SIMV to TT; PSV 10 to TT), indicating	

			serum Na, K, Ca, Mg						increased sympathetic modulation significant reduction of HF (nu), RMSSD and SD1 components during the transition from PSV 10 to the T-piece.	
Hisiu-Nien Shen et al. P Obs 2003	24 ICU	24+	Tracheostomies, frequent cardiac arrhythmias	YES	30 min PSV	Pre-SBT A/C Pre-SBT PSV SBT	*Frequency Domain Measures:* -LF -HF -TP -LF/HF	No significant differences comparing FG and SG and shifting from A/C to PSV.	In the SG, changes were insignificant among the three phases. In the FG, HRV components HF, LF and TP significantly decreased from PSV to SBT.	

10% WP = Width of the histogram at 10% of the peak of the triangle; 50% WP = Width of the histogram at the 50% of the peak of the triangle; CV = Coefficient of variation (CV = SDRRM/Mean RRi X 100); FG = Failure group; HF = High Frequency; LF = Low Frequency; Mean RRi = Mean R-R interval; MSD = Square root of the mean squared differences of successive intervals; pNN50 = Percentage of successive differences in the R-R intervals, whose absolute value exceeds 50 ms; P OBS = Observational Prospective; PSV = Pressure Support Ventilation; RCT = Randomized Controlled Trial; RMSSD = Square Root of the mean of the sum of squares of differences between adjacent normal R-R intervals; RRi = Interval between consecutive heartbeats; SBT = Spontaneous Breathing Trial; SDANN = Standard Deviation of means of R-R intervals of successive 5-min epochs over the 24-hr data recording; SDNN = Standard Deviation of the Normal R Waves; SDNN index = Mean of the Standard Deviation of all R-R intervals for all 5-min epochs captured; SDRRM = Standard Deviation of R-R mean; SDSD = Standard Deviation of the differences adjacent R-R intervals; SG = Success group; TINN = Width of the histogram distribution measured at the base of a triangle; TV = Width of the histogram at the base; TVIPFM = Time-Varying Integral Pulse Frequency Modulation Model; TT = T Tube (T-piece); VLF = Very Low Frequency.

Heart Rate Variability Indices included in the manuscript: abbreviations and short description

Frequency Domain Measures:

- Very Low Frequency (VLF): Power in VLF range (≤0.04 Hz)
- Low Frequency (LF): Power in LF range (0.04-0.15 Hz)
- High Frequency (HF): Power in HF range (0.15-0.4 Hz)
- LF/HF Ratio: LF [ms^2]/HF[ms^2]
- Total Power: The variance of NN intervals over the temporal segment

Time Domain Measures:

- SDNN: Standard deviation of all NN intervals
- SDANN: Standard deviation of the averages of NN intervals in all 5-minute segments of theentire recording
- RMSSD: The square root of the mean of the sum of the squares of differences betweenadjacent NN intervals
- SDNN index: Mean of the standard deviations of all NN intervals for all 5-minute segments of theentire recording
- SDSD: Standard deviation of differences between adjacent NN intervals
- NN50 count: Number of pairs of adjacent NN intervals differing by more than 50 ms in the entirerecording; three variants are possible counting all such NN intervals pairs or onlypairs in which the first or the second interval is longer
- pNN50: NN50 count divided by the total number of all NN intervals

Geometric Domain Measures:

- 10% WP = Width of the histogram at 10% of the peak of the triangle

- 50% WP = Width of the histogram at the 50% of the peak of the triangle

- TINN = Width of the histogram distribution measured at the base of a triangle

- TV = Width of the histogram at the base.

Overall quality of the evidence for the primary outcome according to the Grading of Recommendations, Assessment, Development and Evaluation (GRADE) guidelines.

Supplementary Table S2. Rating the certainty in evidence for LF HRV in predicting the outcome of the SBT.

Question: Should LF HRV be used as a predictor of weaning from IMV?

GRADE domain	Judgement	Concerns about certainty domains
Methodological limitations of the studies	Five out of 6 studies showed serious risk of bias in at least 1 domain. On the opposite, only 2 studies have low risk of bias. Therefore, we judged the studies to have serious methodological limitations	Serious
Indirectness	The patients, intervention and comparators in the studies all provide direct evidence to the clinical question. Slightly differences can be found in SBT and weaning protocol or in definition of ready-to-wean patients. We also noted variability in patients' selection and exclusion criteria. We judged the evidence to have borderline indirectness.	Borderline
Imprecision	Total number of patients was 307, with ample variation though only 1 study were over 100 patients (101). We also noted a great heterogeneity of results.	Serious
Inconsistency	The direction and magnitude of effect varied little across the different studies.	Not serious
Publication bias	Publication bias was not strongly suspected.	Not serious

Outcome	Effect	Number of participants (studies)	Certainty in the evidence
Weaning from IMV	Only one study did not report any association.	299 (6)	LOW<br>⊕⊕OO<br>Due to serious risk of bias.

Supplementary Table S3. Rating the certainty in evidence for HF HRV in predicting the outcome of the SBT.
Question: Should HF HRV be used as a predictor of weaning from IMV?

GRADE domain	Judgement	Concerns about certainty domains
Methodological limitations of the studies	Five out of 6 studies showed serious risk of bias in at least 1 domain. On the opposite, only 2 studies have low risk of bias. Therefore, we judged the studies to have serious methodological limitations	Serious
Indirectness	The patients, intervention and comparators in the studies all provide direct evidence to the clinical question. Slightly differences can be found in SBT and weaning protocol or in definition of ready-to-wean patients. We also noted variability in patients' selection and exclusion criteria. We judged the evidence to have borderline indirectness.	Borderline
Imprecision	Total number of patients was 307, with ample variation though only 1 study were over 100 patients (101). We also noted a great heterogeneity of results.	Serious
Inconsistency	The direction and magnitude of effect varied little across the different studies.	Not serious
Publication bias	Publication bias was not strongly suspected.	Not serious

Outcome	Effect	Number of participants (studies)	Certainty in the evidence
Weaning from IMV	Only one study did not report any association.	299 (6)	LOW<br>⊕⊕OO<br>Due to serious risk of bias.

Supplementary Table S4. Rating the certainty in evidence for LF/HF in predicting the outcome of the SBT.
Question: Should LF/HF be used as a predictor of weaning from IMV?

GRADE domain	Judgement	Concerns about certainty domains
Methodological limitations of the studies	Four out of 6 studies showed serious risk of bias in at least 1 domain. On the opposite, only 2 studies have low risk of bias. Therefore, we judged the studies to have serious methodological limitations	Serious
Indirectness	The patients, intervention and comparators in the studies all provide direct evidence to the clinical question. Slightly differences can be found in SBT and weaning protocol or in definition of ready-to-wean patients. We also noted variability in patients' selection and exclusion criteria. We judged the evidence to have borderline indirectness.	Borderline
Imprecision	Total number of patients was 240, with ample variation though only 1 study were over 100 patients (101). We also noted a great heterogeneity of results.	Serious
Inconsistency	The direction and magnitude of effect varied little across the different studies.	Not serious
Publication bias	Publication bias was not strongly suspected.	Not serious

Outcome	Effect	Number of participants (studies)	Certainty in the evidence
Weaning from IMV	Two studies did not report any association.	232 (5)	LOW<br>⊕⊕OO<br>Due to serious risk of bias and low sample size.

Supplementary Table S5. Rating the certainty in evidence for TP HRV in predicting the outcome of the SBT.
Question: Should TP HRV be used as a predictor of weaning from IMV?

GRADE domain	Judgement	Concerns about certainty domains
Methodological limitations of the studies	Three out of 5 studies showed serious risk of bias in at least 1 domain. On the opposite, only 2 studies have low risk of bias. Therefore, we judged the studies to have serious methodological limitations	Serious
Indirectness	The patients, intervention and comparators in the studies all provide direct evidence to the clinical question. Slightly differences can be found in SBT and weaning protocol or in definition of ready-to-wean patients. We also noted variability in patients' selection and exclusion criteria. We judged the evidence to have borderline indirectness.	Borderline
Imprecision	Total number of patients was 221, with ample variation though only 1 study were over 100 patients (101). We also noted a great heterogeneity of results.	Serious
Inconsistency	The direction and magnitude of effect varied little across the different studies.	Not serious
Publication bias	Publication bias was not strongly suspected.	Not serious

Outcome	Effect	Number of participants (studies)	Certainty in the evidence
Weaning from IMV	Only one study did not report any association.	213 (4)	VERY LOW ⊕OOO Due to serious risk of bias and low sample size.

Supplementary Table S6. Rating the certainty in evidence for VLF HRV in predicting the outcome of the SBT.
Question: Should VLF HRV be used as a predictor of weaning from IMV?

GRADE domain	Judgement	Concerns about certainty domains
Methodological limitations of the studies	One of the 2 studies showed serious risk of bias in at least 1 domain. The other showed a moderate overall risk of bias Therefore, we judged the studies to have serious methodological limitations	Serious
Indirectness	The patients, intervention and comparators in the studies all provide direct evidence to the clinical question. We judged the evidence to have not serious indirectness.	Not serious
Imprecision	Total number of patients was 168 (101 and 67).	Serious
Inconsistency	The direction and magnitude of effect varied little across the different studies.	Not serious
Publication bias	Publication bias was not strongly suspected.	Not serious

Outcome	Effect	Number of participants (studies)	Certainty in the evidence
Weaning from IMV	No clear association was reported.	168 (2)	VERY LOW ⊕OOO Due to serious risk of bias and lack of evidence.

Supplementary Table S7. Rating the certainty in evidence for SDNN in predicting the outcome of the SBT.
Question: Should SDNN HRV be used as a predictor of weaning from IMV?

GRADE domain	Judgement	Concerns about certainty domains
Methodological limitations of the studies	The 4 studies showed serious risk of bias in at least 1 domain. Therefore, we judged the studies to have serious methodological limitations	Serious
Indirectness	The patients, intervention and comparators in the studies all provide direct evidence to the clinical question. Slightly differences can be found in SBT and weaning protocol or in definition of ready-to-wean patients. We also noted variability in patients' selection and exclusion criteria. We judged the evidence to have borderline indirectness.	Serious
Imprecision	Total number of patients was 196, with ample variation.	Serious
Inconsistency	The direction and magnitude of effect varied little across the different studies.	Not serious
Publication bias	Publication bias was not strongly suspected.	Not serious

Outcome	Effect	Number of participants (studies)	Certainty in the evidence
Weaning from IMV	Two studies didn't report any association.	196 (4)	VERY LOW<br>⨁OOO<br>Due to serious risk of bias and low sample size.

Supplementary Table S8. Rating the certainty in evidence for RMSSD HRV in predicting the outcome of the SBT.
Question: Should RMSSD HRV be used as a predictor of weaning from IMV?

GRADE domain	Judgement	Concerns about certainty domains
Methodological limitations of the studies	The 3 studies showed serious risk of bias in at least 1 domain. Therefore, we judged the studies to have serious methodological limitations	Serious
Indirectness	The patients, intervention and comparators in the studies all provide direct evidence to the clinical question. Slightly differences can be found in SBT and weaning protocol or in definition of ready-to-wean patients. We also noted variability in patients' selection and exclusion criteria. We judged the evidence to have serious indirectness.	Serious
Imprecision	Total number of patients was 178 (43+68+67).	Serious
Inconsistency	The direction and magnitude of effect varied little across the different studies.	Not serious
Publication bias	Publication bias was not strongly suspected.	Not serious

Outcome	Effect	Number of participants (studies)	Certainty in the evidence
Weaning from IMV	Only one study reported an association.	178 (3)	VERY LOW<br>⊕OOO<br>Due to serious risk of bias and low sample size.
